# Neuromuscular characteristics of agonists and antagonists during maximal eccentric knee flexion in soccer players with a history of hamstring muscle injuries

**DOI:** 10.1371/journal.pone.0277949

**Published:** 2022-12-01

**Authors:** Ramona Ritzmann, Sarah Strütt, Ignacio Torreno, Janine Riesterer, Christoph Centner, Luis Suarez-Arrones

**Affiliations:** 1 Department of Sports and Sport Science, University of Freiburg, Freiburg, Germany; 2 Praxisklinik Rennbahn AG, Muttenz, Switzerland; 3 FC Basel, Basel, Switzerland; 4 FC Lugano, Lugano, Switzerland; 5 Department of Sports and Computer Science, Section of Physical Education and Sports, Universidad Pablo de Olavide, Seville, Spain; Ritsumeikan University, JAPAN

## Abstract

**Background:**

Muscle strain injuries (MSIs) in the hamstrings are among the most prevalent injuries in elite soccer. We aimed to examine the relation between biomechanical maladaptation in eccentric strength and neuromuscular factors separated by their time and frequency domains.

**Methods:**

20 elite soccer players with a previous history of unilateral MSI in the M. biceps femoris (BF) long head and 20 without MSI participated. Knee flexion torques, rate of torque development (RTD) and electromyographic signals (EMG) of the BF, the M. semitendinosus (SMT) and knee extensors were obtained during unilateral maximal eccentric knee flexions performed at slow (30°/s) and fast (120°/s) angular speeds. Root mean squares and mean power frequency (MF) was calculated.

**Results:**

In the group with a history of MSI, reduced maximal eccentric flexion torque (slow eccentrics -8±11, p<0.05; fast eccentrics -18±13 N*m, p<0.05) and RTD (-33±28 N*m/s, p<0.05; -95±47 N*m/s, p<0.05) concomitantly occurred with diminished agonistic myoelectrical activities (-4±5% of MVC, p<0.05; -10±7% of MVC, p<0.05) and MFs (-24±13 Hz, p<0.05; -24±18 Hz, p<0.05) in the BF. Simultaneously, antagonistic myoelectric activity was elevated (+4±3% of MVC, p<0.05; +3±3% of MVC, p<0.05) in MSI affected legs as compared to unaffected legs for both eccentric contractions. Deficits in myoelectrical activity (r^2^ = 0.715, p<0.05; r^2^ = 0.601, p<0.05) and MF (r^2^ = 0.484, p<0.05; r^2^ = 0.622, p<0.05) correlated with deficits in maximal torque in the affected leg in the MSI group. Analysis of SMT demonstrated no significant differences.

**Conclusion:**

Positive relationships between neuromuscular deficits and the reduced eccentric strength profile underpin neuronal inhibition after MSI. This persistent involvement of dysfunctional synergist and antagonist neural hamstring function in strength weakness is of clinical relevance in sports medicine for prevention and rehabilitation.

## Background

Muscle strain injuries (MSIs) in the hamstrings are among the most prevalent non-contact injuries in soccer [1]. The biceps femoris long head (BF) is the most commonly injured muscle accounting for 60–85% of all muscle injuries [2]. A typical MSI is characterized by sudden pain in the posterior thigh which is elicited by a mechanical disruption of sarcomeres induced by an external eccentric strain exceeding tissue strain capacity [1]. MSI severity is classified into three grades: minor microscopic tearing and some loss of function (grade I), extensive damage, with numerous muscle fibers torn (grade II) and a full (grade III). MSIs among grades I- III in elite sports cause a remarkable time loss of three to 20 weeks from training and competition [3] which results in a diminished athletic performance [4] and subsequently, in financial loss. In the English premier soccer league, the projected loss of output due to such injuries amounted up to 100 Mio $ per season [5].

Despite extensive research to identify risk factors predisposing athletes to MSIs, epidemiological data obtained from team sports across 20 years of research indicates that rates and recurrences of MSIs have not declined in the last decade [6]. Despite the awareness for important risk factors (e.g., previous MSI, short fascicles) [7], the recurrence rates in MSIs are not regressing which highlights that current approaches aiming at the prevention MSIs require further scientific investigation [6–9].

Whilst it is commonly accepted that the etiology of MSIs and its recurrencies are multifactorial in nature, the potential role of the nervous system (NS) has been overlooked and moved into the fore just in the most recent years. The NS regulates muscle length and controls for tolerable changes in myo-elongation with contraction forces achieved by electromechanical coupling [10, 11]. Sensory feedback from myoreceptors collect information about the actual muscle tensions and thus, can protect the muscle from experiencing excessive strain [12]. Some studies point out relevant neuromuscular aspects such deteriorated contributions of biceps femoris and its synergists [13] and chronic activation deficits of hamstrings with a former MSI during contractions. In addition, previously injured hamstrings were reported to be weaker and BF myoelectrical activity lower as compared to the contralateral uninjured hamstring [14, 15]. Pathophysiologically, this is particularly interesting as a previous MSI has been consistently identified as a primary risk factor which predisposes athletes to a secondary MSI [10].

Within a sport specific context, clinicians and trainers integrate eccentric muscle strength as a measure of neuromuscular integrity as a key criterion to determine if an athlete is ready for return to sport or competition after MSI [7, 9]. Measurement paradigms encompass (sub)maximal eccentric isokinetic, leg curls and Nordic hamstring exercises at various speeds (30°/s– 240°/s) [16]. Isokinetic dynamometry is in the top three tests for player screening in elite sport. Joint torques and impulses are commonly compared between the injured and the uninjured legs to detect (a)symmetries. Although there is currently no uniform cut-off for a safe return to sport, an inter-limb difference (injured vs- uninjured) of 10% is frequently used as a consensus [17] without consideration of the neuromuscular factors [18].

Therefore, this cross-sectional study examines the relation between maladaptation in biomechanics and neuromuscular control of eccentric knee flexion separated by their time and frequency domains in a cohort of elite soccer players a previous MSI in the BF long head as compared to healthy controls. Understanding these factors may interact based on myoelectrical and torque-time data, we also propose a tentative hypothesis as to how previous MSIs may lead to neuronal maladaptation of the BF which may dysregulate the force generating capacity.

## Methods

### Experimental design

We executed a cohort study with elite soccer players in a cross-sectional repeated measures design. Players with a previous unilateral MSI in the BF long head were compared to healthy players without MSI. We experimentally evaluated the effect of eccentric loading of the active hamstring muscle tendon unit induced by isokinetic measures on neuromuscular correlates and joint torque among the active range of motion.

The study had been approved by Swiss Ethics for Ethics in Human Experimentation (No EKNZ 2017–01825). The study is registered with the German Register of Clinical Studies (No DRKS00020210).

### Participants

Eighty-seven elite male soccer athletes volunteered to participate in the study. All subjects gave their written informed consent for these experiments.

**Fig 1:**
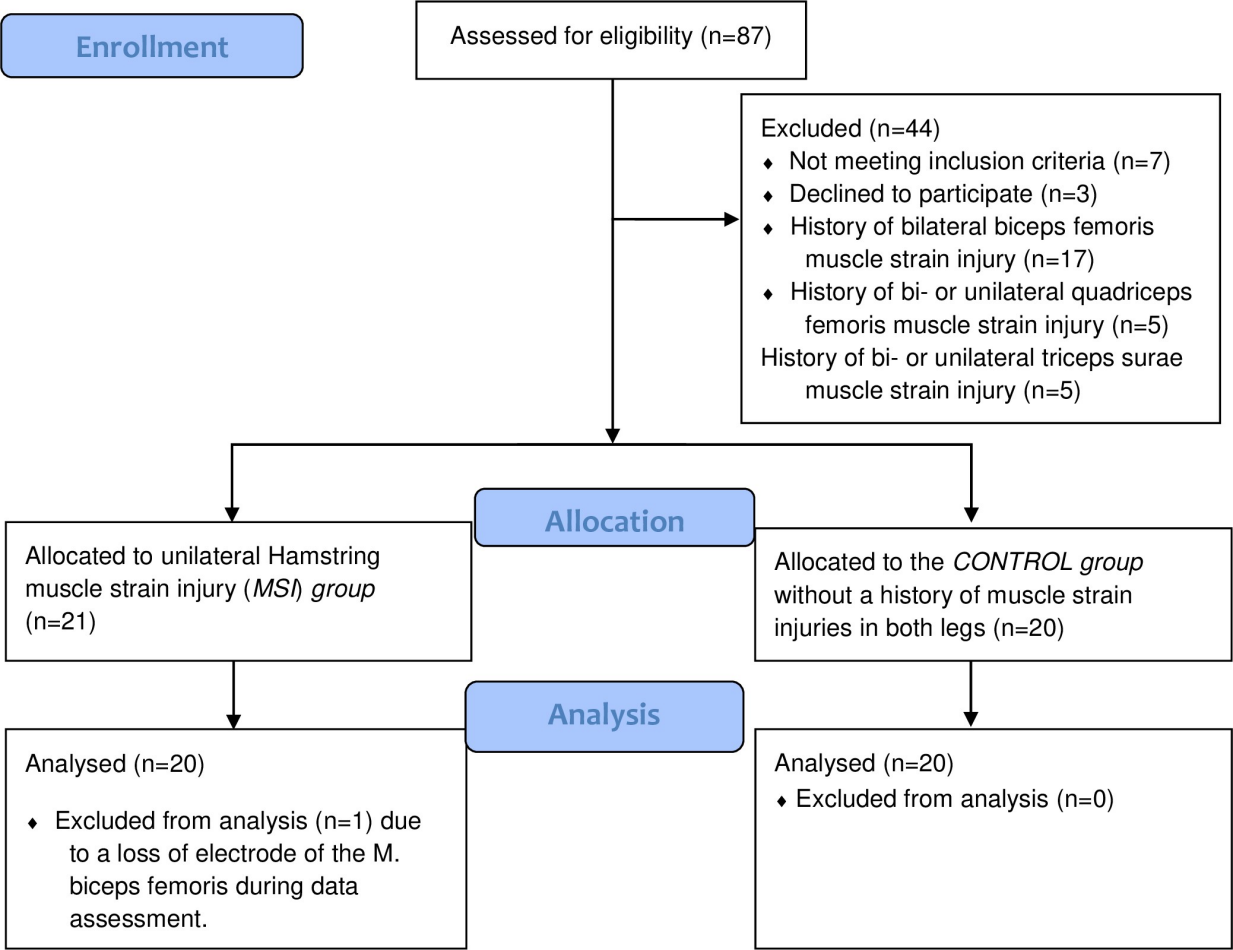
CONSORT flow chart diagram. Enrollment procedures and sample size.

Inclusion criteria were i) an age > 17y, ii) a former traumatic MSI in the BF long head experienced during soccer training or competition certified to be grade I or II by an MRI or MRI/ultrasonography, iii) clinically released, fully participating in regular trainings and matches and elite athletes. Elite refers as a minimum to national selection actively participating in national and international championships. Exclusion criteria were sickness, inflammation, flue, neurologic diseases, acute orthopedic or muscle injuries of the lower extremities, vestibular or proprioceptive dysfunctions, previous cartilage or ligamentous surgeries on the knee joint, neuro-degenerative diseases or single events associated with neural dysfunctions. Participants underwent a medical screening to verify eligibility for the experiment. In this context, subjects were screened for previous hamstring strain injuries [19] and assigned into one of two groups: CONTROL group and MSI group. For the MSI group, subjects were only included if the injury was one-sided and occurred within the last year. A total of n = 20 participants were enrolled in the MSI group and n = 20 participants were enrolled in the CONTROL group (Fig 1).

A priori, the sample size was estimated by means of a power analysis based on a previously executed pilot study including 7 subjects (f = 0.90; alpha = 0.05; power = 0.90).

### Clinical data

The history of muscle strain injuries was addressed by the following items: the affected leg (left or right) and topographic location differentiated by the damaged muscles. The injury event was described by the date, season (pre or competitive season), the activity during which the injury occurred (run, sprint, cut, shot, jump; with or without ball; opponent contact or no opponent contact). Injury severity was addressed by the duration of time loss until full participation in training and competition [20] and the number of competitive games missed. Information about the players`position, training, and match exposure prior to injury were collected.

### Isokinetic dynamometry

Maximal eccentric knee flexion torque was measured using an isokinetic dynamometer (Humac®/NormTM, CSMi, Stoughton, Massachusetts, US) [21]. Measurements were performed in the Praxisklinik Rennbahn AG, (Muttenz, Switzerland) either 10 am– 11:30 am, 3pm - 6pm during the week except for days before and after the matches.

Reliability and validity of this dynamometer for isokinetic concentric knee flexion and procedure are described elsewhere [16, 22]. Subjects were seated in a rigid chair and firmly strapped at the hip, the torso and distal thigh at 90° [23]. The rotational axis of the dynamometer was aligned to the lateral femoral epicondyle, and the lower leg was attached to the dynamometer lever arm above the medial malleolus, with no fixation of the ankle joint. Both legs were tested separately and in a random order; maintaining complete randomness of trials was achieved by flipping a coin. The measurements were preceded by 10 min of warm-up at a stationary cycle ergometer (50W), followed by six submaximal familiarization trials with instructions for maximal contractions and moment cycles in the dynamometer.

Maximum voluntary concentric and eccentric hamstring contractions were randomly performed during slow and fast knee movements with visual feedback for the contraction cycle on a screen in front of the athlete. Counting backwards 3-2-1-0 indicated the beginning of the contraction. According to literature we used knee joint angular velocities at 30°/s and 120°/s, respectively [7, 9, 16]. Encouragement was done verbally for each athlete and each of the repetitions in the same way.

Knee joint excursion was from full extension 0° to 90° flexion [24]. Two successive trials were performed, separated by a rest period of at least 30 s to a maximum of 1 min. All recorded torque signals were corrected for the effect of gravity by calibration prior to each trial [25].

Synchronous sampling of the dynamometer strain gauge signal, lever arm position, and EMG signals was performed at 2 kHz analogue-to-digital conversion rate using an external A/D converter. Torque curves were smoothed by using a Butterworth fourth-order zero-lag low-pass filter with a 10 Hz cut-off frequency.

Peak torque and peak torque index were assessed [24]. The index is the time (ms) between onset until the peak torque has been reached. Peak torques were further extracted for the time intervals 0–50 ms, 50–100 ms and 100–200 ms after onset [9] and for the interval 20° - 0° knee extension (full extension, the BF muscle tendon unit and fascicles are most elongated) [23, 26]. Rate of torque development (RTD, Nm/s) was measured as the slope of the MVC torque-time-curve in the time intervals 0–50 ms, 0–100 ms and 0–200 ms [22, 27, 28]. Impulses (Nm*s) were calculated as the area below the MVC torque-time-curve among the entire ROM and in the time intervals 0–50 ms, 0–100 ms and 0–200 ms.

### Electromyographic (EMG) recordings

Bipolar Ag/AgCl surface electrodes (Ambu Blue Sensor P, Ballerup, Denmark; diameter 9 mm, center-to-center distance 34 mm) were placed over the Musculus (M.) biceps femoris long head (BFLH), the M. semitendinosus (ST), the vastus medialis (VM) and the M. rectus femoris (RF) of the right and left leg according to SENIAM [29]. The longitudinal axes of the electrodes were in line with the direction of the underlying muscle fibers. Inter electrode resistance was kept below 2.5 kΩ by means of shaving, light abrasion, degreasing, and disinfection of the skin. Electrodes and preamplifiers were carefully taped to the skin and the upper and lower leg was covered with a surgical net to avoid electrode movement. EMG Signals were transmitted wireless (Myon, Schwarzenberg, Switzerland), recorded with 2 kHz, amplified, and filtered (band-pass filter 10 Hz– 1 kHz, 1000). Electromyographic data recording was synchronized to isokinetic data.

**Fig 2:**
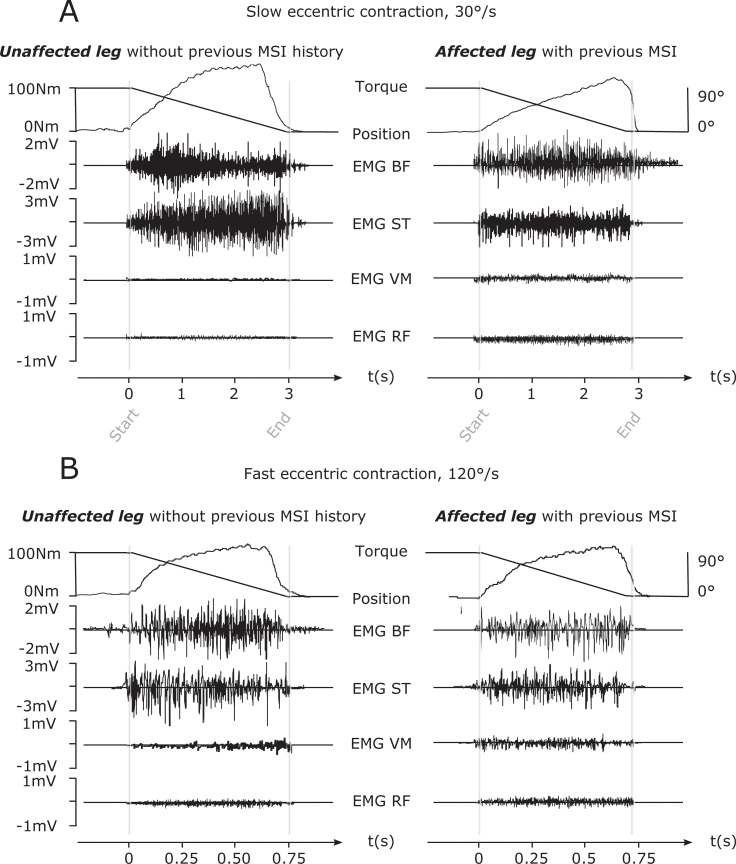
Raw tracings of isokinetic knee torque and electromyogram (EMG). They obtained in a male elite soccer player in the paradigm of maximal eccentric knee flexion contraction during joint movements performed at slow (***A***) and fast (***B***) joint angular speeds (30°/s and 120°/s, respectively) illustrated for the unaffected leg (left) and the affected leg with a history of MSI. Range of joint motion was from 90° to 0° (0° = full knee extension). Upper tracings display the joint torque and position signal. Lower tracings display raw EMG signals recorded from the biceps femoris (BF), semitendinosus (ST), vastus medialis (VM), and rectus femoris (RF) muscles. EMG was lower in the MSI affected compared with the unaffected leg, suggesting significant neural inhibition after MSI. Note the appearance of large EMG amplitude spikes separated by short inter spike periods of high neuromuscular activity in the BF and SMT of the unaffected leg as compared to the affected leg. Furthermore, torque and EMG amplitudes were diminished on the MSI affected side, especially during eccentric and fast concentric contractions (***B***).

The trial with highest impulse (i.e., largest torque-angle curve area) was identified in each subject and selected for further analysis [22]. Fig 2 shows representative torque-time curves and the EMG-time curve of one subject. Data processing was performed according to the gold standard for eccentric torque and EMG as follows:

EMG signals were smoothed by using a linear envelope, which consisted of digital high-pass filtering at a 5-Hz cut-off frequency followed by full-wave rectification and subsequent low-pass filtering at a 10-Hz cut-off frequency [22]. For between group comparison with reference to the MSI history, the BF and SMT root mean squares (RMS) were calculated for equivalent time intervals as for torque measures. For VM and RF, the RMS procedure was applied for 90° - 0° and for 20° - 0°.

$$\mathrm{RMS}=\sqrt{\left(\frac{1}{T}\overset{T}{\underset{0}{\int }}x^{2}(t)dt\right)}$$

where x(t) is the EMG signal and T is the acquisition time. Data were normalized to RMS values obtained during isometric MVC for BF at 20°, SMT at 100° [30], VM and RF at 100° [31] over a period of 500ms.

The spectral parameters were evaluated by standard Fast Fourier Transformation and the median frequency (MF) calculated as follows:

$$\mathrm{MF}=\overset{f_{\mathrm{med}}}{\underset{0}{\int }}P(f)df=\overset{\infty }{\underset{f_{\mathrm{med}}}{\int }}P(f)df=\frac{1}{2}\overset{\infty }{\underset{0}{\int }}P(f)df$$

Where P(f) is the power spectrum density. The MDF, which is the frequency value that divides the spectrum into two regions of equal power, is determined by the overall action potential conduction velocity of the muscle. Analyses were performed using Matlab version 7.12 software (MathWorks, R2011a, Natick, MA, USA).

### Statistics

The legs of the control group were grouped by left and right. The effect of a previous BF MSI on the variables torque, RTD, impulse, the EMG activity (integrals and median frequency) of BF, ST, VM and RF as well as VM/BF co-activation was evaluated using a two-factor analysis of variance (ANOVA): group [CONTROL vs. MSI] and leg [affected vs. unaffected]. The normality of the data was evaluated using Kolmogorov-Smirnov test and all data followed a normal distribution. If the assumption of sphericity, as measured via Mauchly’s test, was violated, the Greenhouse-Geisser correction was used. To correct for multiple testing, the false discovery rate was controlled according to the Benjamini–Hochberg–Yekutieli method. Benjamini–Hochberg–Yekutieli conceptualizes the rate of type I errors [32]. Partial Eta squared (η^2^_p_) was used as an estimate of the effect size for the ANOVA (η^2^_p_ ≤ 0.06 small, 0.06 < η^2^_p_ < 0.14 medium, η^2^_p_ ≥ 0.14 large effect size) [33].

A linear regression was calculated for differences between the unaffected leg and the affected leg with MSI history for neuromuscular and biomechanical parameters. Data were illustrated as scatter plots with regression lines.

All analyses were executed using SPSS 27.0 (SPSS, Inc., Chicago, IL, USA). The values are presented as mean ± standard deviations (*M* ± SD).

## Results

In the MSI group, all hamstring MSIs were traumatic with an acute onset. 95% of MSIs occurred in noncontact situations, only 5% were induced during foul play while in physical contact with opponents. Athletes experienced 55% of MSIs during high-speed running or sprinting, 30% during the change of direction and 15% during jumps. Days of absence from training after MSIs were 48 ± 53 and amounted up 64 ± 81 for absence from competition which resulted in 7 ± 3 missed games. The event of MSI dated back 201 ± 96 days. Anthropometrics, age attributes, training or match exposure are illustrated in Table 1.

**Table 1:** Cohort characteristics, anthropometric data, and athletic specification.

*Groups*	*MSI group*	*CONTROL group*
Number	n = 20	n = 20
UEFA Europe League	n = 12	n = 7
National elite level	n = 8	n = 13
***Position*, *training*, *and match exposure***		
Striker	n = 4	n = 5
Midfielder	n = 3	n = 4
Winger	n = 7	n = 5
Defender	n = 5	n = 4
Goalkeeper	n = 1	n = 2
Total exposure h/player/season (*M* ± SD)	258±89	256±90
Training exposure, h/player/season (*M* ± SD)	218±78	211±81
Match exposure, h/player/season (*M* ± SD)	37±23	39±21
***Anthropometrics***		
Age (years, *M* ± SD)	24±5	24±3
Body mass (kg, *M* ± SD)	75±7	74±8
Stature (cm, *M* ± SD)	179±6	178±6
Injured leg eq. dominant leg	n = 9	n.a.

### Knee flexion torque

Grand means and statistics are illustrated in S1 and S2 Tables.

**Fig 3:**
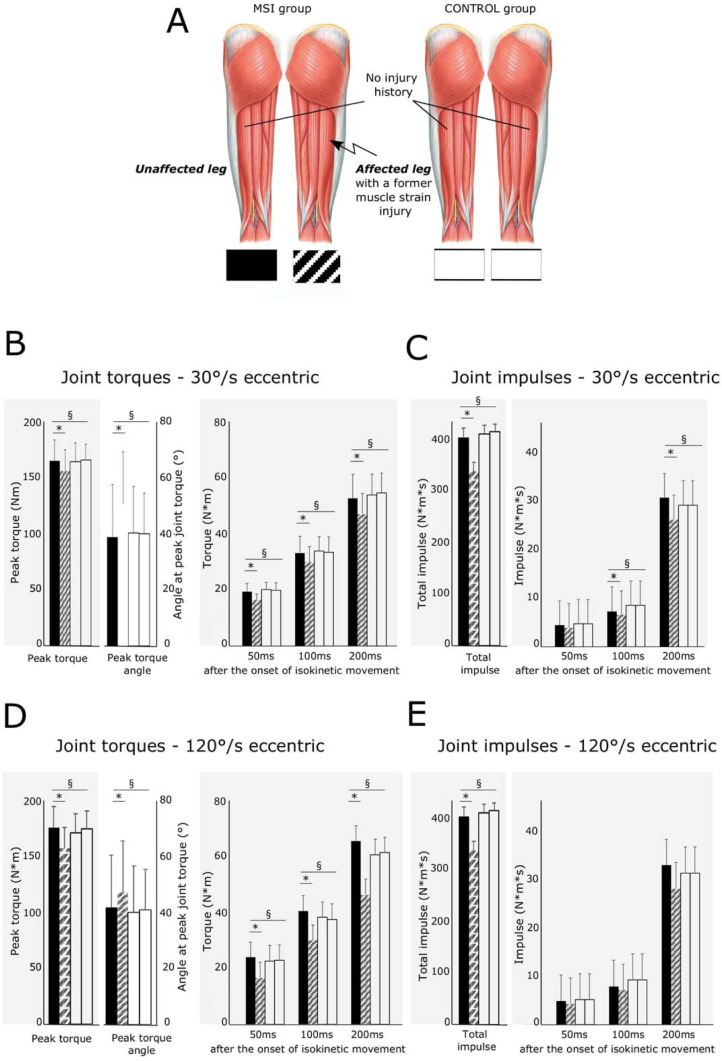
***A*** illustrates the MSI group with previous unilateral hamstring strain injuries experienced in the M. biceps long head (left) and the healthy CONTROL group with bilateral healthy legs. Graphs ***B–E*** illustrate changes in joint torques and impulses: ***B*** displays differences in the peak knee flexion joint torque (ordinate) and the corresponding knee flexion angle as well as the joint torques at 30ms, 50ms 100ms and 200ms after the movement onset of the lever arm at 30°/s speed for both groups. Graph ***C*** displays differences in the total impulse and impulses calculated for the intervals 0–50ms, 0–100ms and 0–200ms after movement onset of the lever arm at 30°/s speed. Joint torques at 120°/s are illustrated in graph ***D***. Joint Impulses at 120°/s are illustrated in graph ***E***. Values are means ± SD; § indicates significant group * leg interaction effects; * symbolizes significant pairwise differences.

For the slow eccentric contraction, the ANOVA revealed a significant interaction (group × leg) effect for the peak torque, peak torque index, peak torque_100ms_ and peak torque_200ms_. Eccentric knee flexor torques were reduced and the peak torque index was increased in the leg with a history of MSI (Fig 3). For RTD_100_, RTD_200_ and Impulse_50_, the analysis showed a significant interaction of group × leg. These parameters were significantly reduced in the leg with an MSI history in the MSI group. Effect sizes ranged from medium to large.

Likewise, for the fast eccentric contraction, the ANOVA revealed a significant interaction (group × leg) effect for each of the parameters collected knee flexor torques and RTD. The impulse and Impulse_200ms_ showed a significant interaction of group × leg. All parameters were reduced in the leg with an MSI history in the MSI group despite peak torque index. Effect sizes were large.

### EMG activity

**Fig 4:**
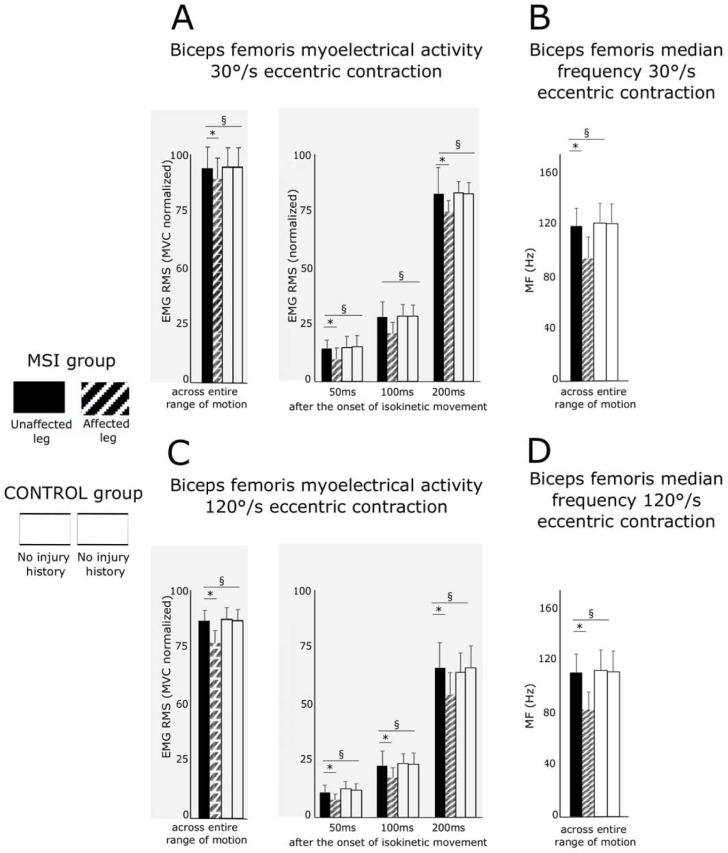
Grand means of myoelectrical activities. ***A*** illustrates Biceps femoris myoelectrical activity as the root mean square normalized to MVC and ***B*** the median frequency at 30°/s during the eccentric knee flexion for the MSI and CONTROL group. Grands means for myograms are illustrated across the entire range of motion and the intervals 0–50ms, 0–100ms and 0–200ms after movement onset of the lever arm at 30°/s speed. ***C*** illustrates Biceps femoris myoelectrical activity and ***D*** the median frequency at 120°/s during the eccentric knee flexion for the MSI and CONTROL group. Not the reduced myoelectrical activities and frequencies for the affected leg of the MSI groups as compared to the healthy contralateral side or the CONTROL group. Values are means ± SD; § indicates significant group * leg interaction effects; * symbolizes significant pairwise differences.

Grand means and statistics are illustrated in Tables 2 and 3. Graphics are illustrated in Fig 4.

**Table 2:** Neuromuscular activity.

*Eccentric contraction*	*MSI group*		*CONTROL group*		*Statistics*	
	unaffected	MSI affected	unaffected	unaffected	ANOVA	η^2^_p_
***M*. *biceps femoris***						
EMG_RMS_	**95±4**	**91±5** *	96±4	96±4	**F = 7.014, p = 0.01**	0.084
EMG_RMS_ (0–50 ms)	**15±4**	**10±5** *	14±5	15±6	**F = 6.010, p = 0.017**	0.073
EMG_RMS_ (50–100 ms)	29±7	21±7	29±8	30±8	**F = 4.973, p = 0.029**	0.061
EMG_RMS_ (100–200 ms)	**83±11**	**75±9** *	81±8	83±9	F = 3.246, p = 0.076	0.041
EMG_RMS_ (20°–0°)	**90±8**	**81±5** *	89±6	89±6	**F = 9.696, p = 0.003**	0.113
MF (Hz)	**118±14**	**94±16** *	120±15	123±15	**F = 12.600, p = 0.001**	0.142
***M*. *semitendinosus***						
EMG_RMS_	94±5	93±5	94±4	95±4	F = 1.090, p = 0.30	0.014
EMG_RMS_ (0–50 ms)	19±5	18±4	19±3	19±5	F = 0.358, p = 0.551	0.005
EMG_RMS_ (50–100 ms)	27±6	26±6	27±7	27±5	F = 0.167, p = 0.684	0.002
EMG_RMS_ (100–200 ms)	83±8	82±12	83±11	84±9	F = 3.246, p = 0.076	0.041
EMG_RMS_ (20°–0°)	87±6	87±6	88±5	89±7	F = 0.102, p = 0.750	0.001
MF (Hz)	83±10	84±9	82±9	84±9	F = 0.210, p = 0.648	0.003
***M*. *vastus medialis***						
EMG_RMS_	**3±2**	**7±2** *	3±1	4±2	**F = 31.320, p<0.001**	0.292
EMG_RMS_ (20°–0°)	**3±3**	**6±2** *	4±1	3±2	**F = 18.751, p<0.001**	0.198
***M rectus femoris***						
EMG_RMS_	**3±3**	**7±2** *	3±1	3±3	**F = 15.251, p<0.001**	0.167
EMG_RMS_ (20°–0°)	**4±4**	**7±2** *	3±2	4±2	**F = 14.088, p = 0.007**	0.093

Neuromuscular activation [electromyographic activity (root mean square normalized to MVC, EMG_RMS_) and median power frequency (MF)] obtained of knee flexor and extensor muscles during slow (30°/s) eccentric hamstring contractions. Differences in normalized EMG_RMS_ are illustrated for the M. biceps femoris, M. semitendinosus, M. vastus lateralis and M. rectus femoris for the entire range of motion and the interval 20°–0° extension. BF and SMT is displayed for 0–50 ms, 50–100 ms and 100–200 ms intervals as well.

Values are means ± SE.

* indicates a significant difference between legs. P values, F values and effect sizes (η^2^_p_) are given for significant group*leg interaction effects.

**Table 3:** Neuromuscular activity.

*Eccentric contraction*	*MSI group*		*CONTROL group*		*Statistics*	
	unaffected	MSI affected	unaffected	unaffected	ANOVA	η^2^_p_
***M*. *biceps femoris***						
EMG_RMS_	**86±8**	**76±5**	86±5	85±5	**F = 12.750, p = 0.01**	0.144
EMG_RMS_ (0–50 ms)	**11±2**	**8±3**	13±4	12±3	**F = 4.177, p = 0.044**	0.052
EMG_RMS_ (50–100 ms)	**23±5**	**18±4**	24±4	22±5	**F = 6.277, p = 0.014**	0.076
EMG_RMS_ (100–200 ms)	**67±9**	**55±10**	65±9	67±10	**F = 132.654, p<0.001**	0.636
EMG_RMS_ (20°– 0°)	**75±7**	**64±8**	75±8	74±7	**F = 9.050, p = 0.006**	0.096
MF	103±26*	**79±13**	109±15	108±15	**F = 61.097, p<0.001**	0.46
***M*. *semitendinosus***						
EMG_RMS_	85±4	86±5	85±4	86±4	F = 0.193, p = 0.662	0.003
EMG_RMS_ (0–50 ms)	15±5	13±5	14±3	16±4	F = 0.654, p = 0.421	0.009
EMG_RMS_ (50–100 ms)	23±6	22±8	17±6	19±6	F = 0.167, p = 0.684	0.002
EMG_RMS_ (100–200 ms)	72±10	69±13	71±9	71±10	F = 0.182, p = 0.671	0.002
EMG_RMS_ (20°– 0°)	76±8	75±7	75±9	77±8	F = 0.073, p = 0.788	0.001
MF	75±10*	76±9	76±9	77±8	F = 27.070, p = 828	0.001
***M*. *vastus medialis***						
EMG_RMS_	**4±4**	**7±2**	3±2	4±3	**F = 8.227, p = 0.008**	0.106
EMG_RMS_ (20°– 0°)	**4±4**	**8±4**	4±3	4±2	**F = 5.143, p = 026**	0.063
***M rectus femoris***						
EMG_RMS_	**4±4**	**7±2**	3±2	4±2	**F = 1.550, p = 0.061**	0.041
EMG_RMS_ (20°– 0°)	**3±5**	**7±3**	3±2	2±2	**F = 10.127, p = 0.016**	0.099

Neuromuscular activation [electromyographic activity (root mean square normalized to MVC, EMG_RMS_) and median power frequency (MF)] obtained during fast (120°/s) eccentric hamstring contractions. Differences in normalized EMG_RMS_ are illustrated for the M. biceps femoris, M. semitendinosus, M. vastus lateralis and M. rectus femoris for the entire range of motion and the interval 20°–0° extension. BF and SMT is displayed for 0–50 ms, 50–100 ms and 100–200 ms intervals as well.

Values are means ± SE.

* indicates a significant difference between legs. P values, F values and effect sizes (η^2^_p_) are given for significant group*leg interaction effects.

The ANOVA revealed a significant interaction (group × leg) effect for BF EMG_rms_ for both eccentric contraction speeds which indicates a reduced myoelectrical activation in the leg with a history of MSI (Fig 4). For all time intervals, the ANOVA revealed statistical significance despite for 100–200 ms after contraction onset in the slow contraction. ANOVA results for SMT EMG_rms_ revealed no significant differences. Effect sizes were medium to large.

For the antagonistic knee extensors VM and RF, the ANOVA revealed a significant interaction (group × leg) effect for both contraction speed in all time intervals (Tables 2 and 3).

### EMG frequency

The ANOVA revealed a significant interaction (group × leg) effect for BF MF in the slow and fast eccentric condition (Tables 2 and 3). Results for SMT MF revealed no significant differences. Effect sizes were large.

### Co-contraction RF/BF-ratio and VM/BF-ratio

Grand means are illustrated in Table 4. The ANOVA revealed a significant interaction (group × leg) effect for the co-contraction ratios in the slow eccentric contraction and the fast eccentric indicating an increase in antagonistic co-contraction in the affected leg with MSI history as compared to the unaffected leg. Effect sizes ranged from medium to large.

**Table 4:** Co-contraction.

*Eccentric contraction*	*MSI group*		*CONTROL group*		*Statistics*	
	unaffected	MSI affected	unaffected	unaffected	ANOVA	η^2^_p_
***Slow angular speed (30°/s)***						
RF/BF-ratio	**0.02±0.01**	**0.05±0.03**	0.01±0.01	0.02±0.01	**F = 4.550, p = 0.04**	0.144
VM/BF-ratio	**0.03±0.02**	**0.06±0.05**	0.03±0.02	0.04±0.02	**F = 10.026, p = 0.02**	0.179
***Fast angular speed (120°/s)***						
RF/BF-ratio	**0.03±0.02**	**0.07±0.03**	0.02±0.03	0.03±0.03	**F = 7.991, p = 0.013**	0.082
VM/BF-ratio	**0.03±0.02**	**0.06±0.05**	0.03±0.02	0.04±0.02	**F = 12.718, p = 0.003**	0.198

### Regressions

**Fig 5:**
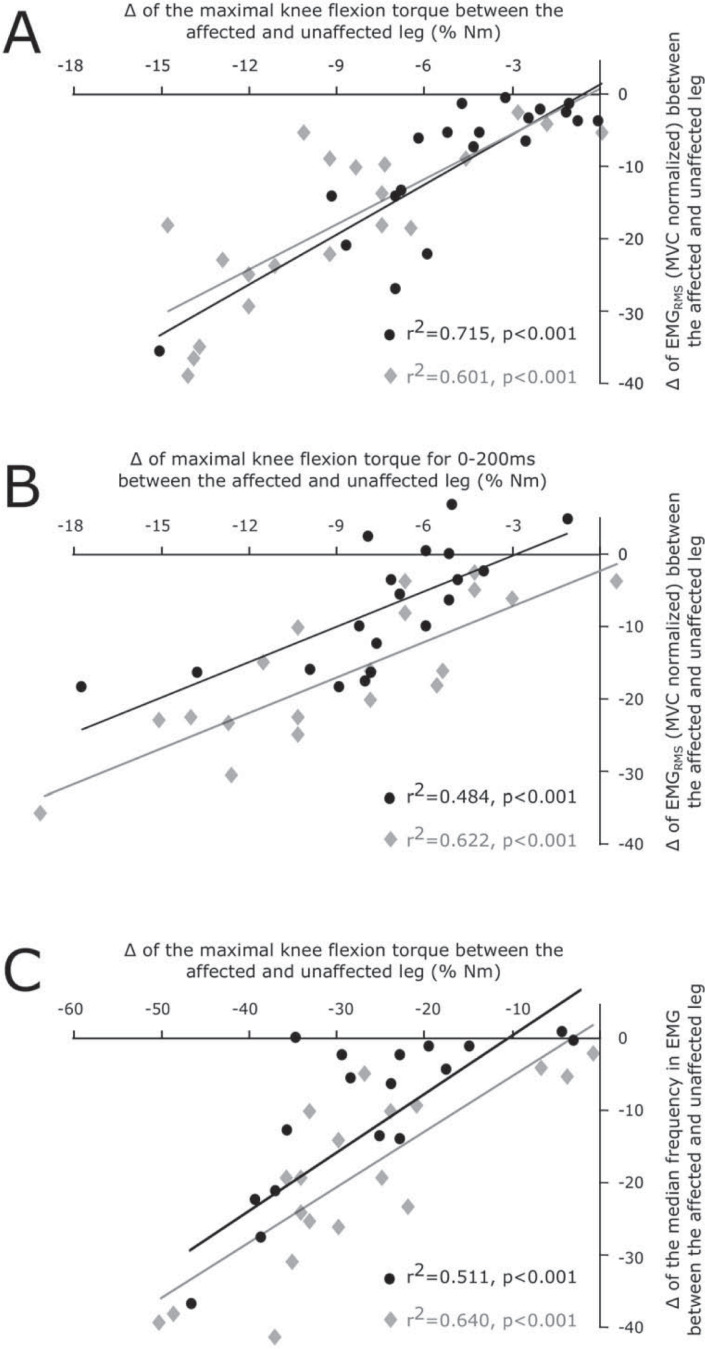
Scatter plots with regression lines. They are illustrated for differences (Δ) between the unaffected leg and the affected leg with MSI history in the biceps femoris long head (BF) muscles of the MSI group (n = 20) for the slow (30°/s, black) and fast (120°/s, grey) eccentric contraction. ***A*** illustrates the plot for maximal torque and BF EMG_RMS_ (normalized to MVC) for the entire range of motion. ***B*** illustrates maximal torque and BF EMG_RMS_ for the interval 0–200ms after contraction onset. Graph ***C*** illustrates the plot for maximal torque and the corresponding the medium frequency. Regressions ***A–C*** are significantly positive.

Scatter plots with regression lines are illustrated in Fig 5. The statistical analysis revealed positive regressions for normalized BF EMG_rms_ and maximal knee flexion torque (slow eccentrics F = 48.648, p<0.001, r^2^ = 0.715 and fast eccentrics F = 29.566, p<0.001, r^2^ = 0.601). BF EMG_rms_ and maximal knee flexion torque 0–200 ms after contraction onset and for BF MF (slow eccentrics F = 18.833, p<0.001, r^2^ = 0.484 and fast eccentrics F = 38.196, p<0.001, r^2^ = 0.622) and maximal knee flexion torque revealed a positive interrelationship for both contraction speeds (slow eccentrics F = 20.843, p<0.001, r^2^ = 0.511 and fast eccentrics F = 34.724, p<0.001, r^2^ = 0.641), as well.

## Discussion

The current study permits major insights into the neuromuscular mechanisms underlying persistently reduced eccentric force generating capacity of athletes with a history of MSI in the BF. A reduced maximal eccentric knee flexion torque, rate of torque development and impulse during eccentric knee flexion concomitantly occurred with diminished myoelectrical activities and MFs in the BF while antagonistic myoelectric activity was elevated in affected legs with a history of MSI as compared to the unaffected legs. Myoelectric deficits correlated with deficits in torque profiles in the MSI affected leg. Importantly, no differences for the contralateral, non-injured leg of the MSI group and the healthy control group have been observed. These outcomes provide evidence for the interrelationship between neuromuscular parameters and muscle function when sustaining supramaximal eccentric loads.

### Eccentric knee flexion torque

A left shift of the torque-angle-curve derived from slow concentric and fast eccentric knee flexion dynamometry [34] concomitant with a reduced torque generating capacity to sustain supramaximal external forces was observed in legs with a history of a healed previous MSI in the BF long head (Figs 2 and 3). That knee flexors of the affected leg generate their peak torques at shorter muscle lengths than unaffected contralateral knee flexors is in line with previous results obtained in recreational athletes [24]. But it is new and remarkable finding for the population of elite soccer players in the active stage of their careers. Eccentric knee flexion torque was reduced by 5–15% in the leg with a previous MSI. With reference to a bilateral deficit of 10% to allow a safe return to sport after MSI [17], the results are particularly delicate at high angular speeds. Besides maximal torque and impulse (S1 and S2 Tables), the rate of torque development at the beginning of the eccentric movement was diminished. The rate of torque development refers to the ability of the neuromuscular system to increase contractile forces from a resting level as fast as possible [21]. Causal relationships with neuromuscular deficits and the reduced eccentric strength profile underpin dysfunctional synergist and antagonist neural hamstring function [18] as the underlying mechanism as highlighted in detail below.

### Neuromuscular responses

Long-term weakness after musculoskeletal injury can be mediated by both muscular and neural adaptations [1]. Whilst myo-structural maladaptation have been reported previously [24], the impact of a prior hamstring strain injury on neural function has been less understood [13–15]. Particularly, neuromuscular adaptation of elite athletes has been scarcely studied, yet [13]. In the current study with reference to healthy controls, between limb comparisons of normalized neuromuscular factors revealed differences in neural hamstring function between the affected and unaffected limbs (both time and frequency domain, Fig 4). Our current findings manifest a 5% to 30% reduction of myoelectrical activity and frequency during slow and fast eccentric contractions (Table 2 and S1 Table).

With reference to the ***time domain***, the muscles’ EMG activity is related to the extent of the muscle fibers’ recruitment [35] and firing rate [35, 36]. A high EMG activity results from a high number of recruited muscle fibers and high motor unit discharge rates [37] and is accompanied by high forces generated by the musculature [37]. Based on these relations, the EMG activity is the primary indicator for the activation intensity of the BF muscle at least within certain limits such as synergistic cross talk and nonlinearity [38]. A high EMG_RMS_, therefore, is indicative of an increased muscle excitation allowing athletes to maintain myofibrillar linkages and thus active force production during eccentric loading without being excessively stretched or (micro)damaged [22]. A reduced EMG_RMS_, most critical in conditions of maximal muscle lengthening during fast eccentric loading (i.e. at 20° at 120°/s flexion approx. -15%), is indicative of neuronal inhibition, reduced activation intensities and diminished forces [37] leading to an increased risk of unphysiological muscle lengthening during the eccentric loading [18].

This close relationship between myoelectric activity and knee flexion torque is further highlighted by the findings from our regression analysis, which indicate that deficits in EMG amplitude predict 60–71% of the variance in deficits in maximal eccentric knee flexion torques for slow and fast angular velocities, respectively (Fig 5). Although our findings within the laboratory cannot easily be transferred into situation on the field, it should be noted that during sprinting and changes of direction (as primary injury movements), the muscle undergoes precisely this eccentric stretching and an instantaneous high tensile force at high speed [11].

With reference to the ***frequency domain***, the power spectrum of the EMG in the affected BF shifted towards lower frequencies (approx. 25%) for the cohort of elite soccer players with a history of MSI (Tables 2 and 3). Such lower frequency shifting has been associated with changes in firing properties towards diminished motor unit action potential [39], increased activation of slow motor units and a concomitant decrease in activation of fast units [40]. Interestingly, the observed responses confirm persistent neuromuscular deficits when the opposite would be needed to protect the muscle from structural damage.

With reference to the ***augmented antagonistic co-activation*** on the side with a history of an MSI it becomes apparent that not only the neuronal pattern synergistic but also the antagonistic muscles are affected. One of the direct mechanical effects of agonist-antagonistic coactivation is a reduction in the resultant forces and moments [41]. Thus, the increased myoelectrical activity of the quadriceps femoris during eccentric knee flexion (S2 Table) indicates that antagonist muscles provide more opposing force to the contracting hamstrings. Beside the BF, the ST and M. semimembranosus also belongs to the synergic hamstrings. While myoelectrical signals cannot be derived from the M. semimembranosus, ST shows no changes among the entire protocol (Tables 3 and 4). As the rectus femoris and vasti oppose the action of the BF, the eccentric net torque is diminished [42]. A high antagonist coactivation is associated with a protective stabilization throughout voluntary joint stiffening [41] while less antagonist coactivation is associated with a high skill level, fine coordination or familiarity with a movement pattern [42]. Both aspects are important for the clinical implication as the protective safety is an important deliberate neural control strategy in terms of injury avoidance. No differences for the contralateral, non-injured leg of the MSI group and the healthy control group have been observed. This indicates that a neuromuscular and biomechanical maladaptation’s in the leg with a history of MSI is certainly due to the previously strained muscle and not individual-specific.

### Limitations

For a conclusive statement, it is crucial to consider the limitations of the study. Two aspects are of substantial importance: First, we identified the parameters such as myoelectrical amplitude and frequency of the agonists and the ratio of antagonist-agonist myoelectrical activity to affect the eccentric torque produced by the knee flexors. But the extent to which these factors are responsible for the reduction in force cannot be determined with this study protocol. Second, a follow-up describing the long-term effects of reduced neuromuscular capacity and its association with recurrences was not included in the present study. Thus, we cannot make any statement about the extent to which the neuromuscular adaptations are of long-term relevance.

### Clinical consequences

The positive regressions (Fig 5) between maximal torques and BF EMG_RMS_ or MF underpin prolonged neuronal deficits during maximal concentric muscle activation associated with a reduced eccentric strength. Importantly, the athletes of the MSI cohort had already been clinically released and fully participating in regular training and match operations for weeks. Thus, despite the full recovery of the injury, neuronal deficits are still present. The causative between maladaptation in muscle mechanics and neuromuscular risk factors separated by their time and frequency domains (Fig 5) in the cohort of elite soccer players is a notable scientific insight and noteworthy being implemented into sports medical diagnostics and therapy to support advances in recovery. There is an ongoing debate about neuromuscular screening of elite athletes [1, 43], combining dynamometry and electromyography for criteria-based return to sport or return to competition after injury to prevent recurrence due to chronic weakness. Individual intervention-relevant weaknesses could thus be detected and clinically prioritized.

## Conclusion

Since neuronal mediated hamstring weakness appears to be a long-lasting consequence of MSI with reference to our results and a primary predictor for recurrences [44], neuromuscular factors represent important deficits which needs to be considered in clinic and rehabilitation [18]. There is strong evidence to suggest that muscle injuries typically occurs when the muscle is subjected to strain during high tension [45]. Activation deficits require more attention in RTS diagnostics. Further, late rehabilitation including eccentric exercises seems appropriate for this purpose because of its positive effects on muscle activation [46]. Further work is needed to clarify the potential contribution of neuromuscular inhibition to MSI recurrences and to examine the efficacy of rehabilitation protocols on voluntary hamstring activation.

## Supporting information

S1 Table Knee joint torques (N*m), rate of torque development (N*m/s) and contractile impulse (N*m*s) during the slow eccentric contraction (speed 30°/s) illustrated as means ± standard deviations (*M* ± SD). RTD (Δ moment / Δ time) was calculated in time intervals 0–50, 0–100, and 0–200ms (Δ time) from the onset of contraction. Contractile impulse, defined as the area covered by the moment-time curve (∫ moment dt), was calculated in the same time intervals. Values are means ± SE. * indicates a significant difference between legs. P values and effect sizes partial eta square (η^2^_p_) are given for significant group*leg interaction effects. Bolt numbers refer to significant pairwise differences between the affected and non-affected leg.
(DOCX)

S2 Table Knee joint torques (N*m), rate of torque development (N*m/s) and contractile impulse (N*m*s) during the fast eccentric contraction (speed 120°/s) illustrated as means ± standard deviations (*M* ± SD). RFD (Δ moment / Δ time) was calculated in time intervals of 0–50, 0–100, and 0-200ms (Δ time) from the onset of contraction. Contractile impulse, defined as the area covered by the moment-time curve (∫ moment dt), was calculated in the same time intervals. Values are means ± SE. * indicates a significant difference between legs. P values and effect sizes partial eta square (η^2^_p_) are given for significant group*leg interaction effects. Bolt numbers refer to significant pairwise differences between the affected and non-affected leg.
(DOCX)
