# Supplementary material for: Neuromuscular characteristics of agonists and antagonists during maximal eccentric knee flexion in soccer players with a history of hamstring muscle injuries
Source: PLoS One. 2022 Dec 1;17(12):e0277949. doi: 10.1371/journal.pone.0277949 (PMC9714924; doi:10.1371/journal.pone.0277949)
Supplement: S1 Table — RTD (Δ moment / Δ time) was calculated in time intervals 0–50, 0–100, and 0–200ms (Δ time) from the onset of contraction. Contractile impulse, defined as the area covered by the moment-time curve (∫ moment dt), was calculated in the same time intervals. Values are means ± SE. * indicates a significant difference between legs. P values and effect sizes partial eta square (η2p) are given for significant group*leg interaction effects. Bolt numbers refer to significant pairwise differences between the affected and non-affected leg. (DOCX) [file pone.0277949.s001.docx]

| ***Eccentric contraction*** | ***MSI Group*** | | ***Control Group*** | | ***Statistics*** | |
| --- | --- | --- | --- | --- | --- | --- |
|  | unaffected | MSI affected | unaffected | unaffected | ANOVA | η^2^_p_ |
| Peak Torque (N*m) | **165±19** | **157±19*** | 165±17 | 166±14 | **F=5.511, p=0.023** | 0.091 |
| Peak torque index (°) | **51±12** | **39±14*** | 40±11 | 40±13 | **F=4.745, p=0.032** | 0.060 |
| Torque_50ms_  (N*m) | **20±6** | **17±6*** | 20±5 | 20±6 | F=1.222, p=0.272 | 0.016 |
| Torque_100ms_  (N*m) | 34±9 | 31±7 | 34±8 | 34±7 | **F=3.592, p=0.044** | 0.069 |
| Torque_200ms_  (N*m) | **53±11** | **48±10*** | 55±10 | 56±11 | **F=7.913, p=0.017** | 0.095 |
| RTD_50ms_ (N*m/s) | **395±119** | **335±110*** | 409±103 | 404±112 | F=1.624, p=0.206 | 0.021 |
| RTD_100ms_ (N*m/s) | **338±87** | **305±74*** | 345±75 | 339±70 | **F=2.592, p=0.042** | 0.062 |
| RTD_200ms_ (N*m/s) | **267±54** | **238±49*** | 274±48 | 278±56 | F=1.913, p=0.171 | 0.025 |
| Impulse (N*m*s) | **470±33** | **443±61*** | 469±34 | 467±42 | F=1.561, p=0.250 | 0.025 |
| Impulse_50ms_ (N*m*s) | **6±1** | **4±1*** | 5±1 | 5±1 | **F=4.507, p=0.037** | 0.066 |
| Impulse_100ms_ (N*m*s) | 9±2 | 8±2 | 9±2 | 9±3 | F=1.408, p=0.239 | 0.018 |
| Impulse_200ms_ (N*m*s) | **36±8** | **32±8*** | 36±12 | 36±7 | F=0.677, p=0.413 | 0.009 |
