# Supplementary material for: Neuromuscular characteristics of agonists and antagonists during maximal eccentric knee flexion in soccer players with a history of hamstring muscle injuries
Source: PLoS One. 2022 Dec 1;17(12):e0277949. doi: 10.1371/journal.pone.0277949 (PMC9714924; doi:10.1371/journal.pone.0277949)
Supplement: S2 Table — RFD (Δ moment / Δ time) was calculated in time intervals of 0–50, 0–100, and 0-200ms (Δ time) from the onset of contraction. Contractile impulse, defined as the area covered by the moment-time curve (∫ moment dt), was calculated in the same time intervals. Values are means ± SE. * indicates a significant difference between legs. P values and effect sizes partial eta square (η2p) are given for significant group*leg interaction effects. Bolt numbers refer to significant pairwise differences between the affected and non-affected leg. (DOCX) [file pone.0277949.s002.docx]

| ***Eccentric contraction*** |  | ***MSI group*** | ***CONTROL group*** | | ***Statistics*** | |
| --- | --- | --- | --- | --- | --- | --- |
|  | MSI unaffected | MSI affected | unaffected | unaffected | ANOVA | η^2^_p_ |
| Peak Torque (N*m) | **176±19** | **158±19*** | 172±17 | 175±16 | **F=7.786, p=0.007** | 0.093 |
| Peak torque index (°) | 42±12 | 47±10 | 40±11 | 41±9 | F=0.006, p=0.345 | 0.012 |
| Torque_50ms_  (N*m) | **22±6** | **15±5*** | 20±5 | 21±6 | **F=7.685, p=0.007** | 0.092 |
| Torque_100ms_  (N*m) | **37±6** | **27±5*** | 34±8 | 34±7 | **F=9.249, p=0.003** | 0.108 |
| Torque_200ms_  (N*m) | **59±12** | **42±10*** | 55±10 | 55±12 | **F=13.153, p=0.001** | 0.148 |
| RTD_50ms_ (N*m/s) | **430±116** | **300±93*** | 409±103 | 412±116 | **F=7.685, p=0.007** | 0.092 |
| RTD_100ms_ (N*m/s) | **365±61** | **270±54*** | 345±75 | 339±70 | **F=9.249, p=0.003** | 0.108 |
| RTD_200ms_ (N*m/s) | **295±62** | **209±50*** | 274±48 | 277±59 | **F=13.153, p=0.001** | 0.148 |
| Impulse (N*m*s) | **406±61** | **340±52*** | 414±45 | 418±60 | **F=8.386, p=0.005** | 0.099 |
| Impulse_50ms_ (N*m*s) | 5±1 | 4±1 | 5±1 | 5±1 | F=0.399, p=0.529 | 0.005 |
| Impulse_100ms_ (N*m*s) | 7±2 | 7±1 | 8±2 | 9±2 | F=2.332, p=0.131 | 0.030 |
| Impulse_200ms_ (N*m*s) | **31±7** | **26±7*** | 28±6 | 29±6 | **F=4720, p=0.048** | 0.061 |
